# A direct comparison of protein interaction confidence assignment schemes

**DOI:** 10.1186/1471-2105-7-360

**Published:** 2006-07-26

**Authors:** Silpa Suthram, Tomer Shlomi, Eytan Ruppin, Roded Sharan, Trey Ideker

**Affiliations:** 1 Department of Bioengineering, University of California, San Diego, CA 92093, USA; 2 Program in Bioinformatics, University of California, San Diego, CA 92093, USA; 3 School of Computer Science, Tel-Aviv University, Tel Aviv 69978, Israel

## Abstract

**Background:**

Recent technological advances have enabled high-throughput measurements of protein-protein interactions in the cell, producing large protein interaction networks for various species at an ever-growing pace. However, common technologies like yeast two-hybrid may experience high rates of false positive detection. To combat false positive discoveries, a number of different methods have been recently developed that associate confidence scores with protein interactions. Here, we perform a rigorous comparative analysis and performance assessment among these different methods.

**Results:**

We measure the extent to which each set of confidence scores correlates with similarity of the interacting proteins in terms of function, expression, pattern of sequence conservation, and homology to interacting proteins in other species. We also employ a new metric, the Signal-to-Noise Ratio of protein complexes embedded in each network, to assess the power of the different methods. Seven confidence assignment schemes, including those of Bader *et al*., Deane *et al*., Deng *et al*., Sharan *et al*., and Qi *et al*., are compared in this work.

**Conclusion:**

Although the performance of each assignment scheme varies depending on the particular metric used for assessment, we observe that Deng *et al*. yields the best performance overall (in three out of four viable measures). Importantly, we also find that utilizing any of the probability assignment schemes is always more beneficial than assuming all observed interactions to be true or equally likely.

## Background

Systematic elucidation of protein-protein interaction networks will be essential for understanding how different behaviors and protein functions are integrated within the cell. Recently, the advent of high-throughput experimental techniques like yeast two-hybrid (Y2H) assays [1] and co-immunoprecipitation (co-IP) screens [2] has led to the elucidation of large-scale protein interaction networks in different species, including *S. cerevisiae *(yeast) [2-5], *D. melanogaster *(fly) [6], *C. elegans *(worm) [7] and *H. sapiens *(human) [8,9]. These networks, while incorporating thousands or tens of thousands of measured interactions, have so far only partially covered the complete repertoire of protein interactions in an organism, and they have been determined to contain a significant number of false-positive interactions depending on the study [10]. However, recent years have also seen an increase in the accumulation of other sources of biological data such as whole genome sequence, mRNA expression, protein expression and functional annotation. This is particularly advantageous as some of these data sets can be utilized to reinforce true (physical) protein interactions while downgrading others. For instance, biologically relevant protein interactions have been shown to have high mRNA expression correlation for the proteins involved [11].

As a result, many integrative bioinformatic approaches have been developed to unearth true protein-protein interactions. These can be mainly divided into two categories: (1) methods that assign reliability measurements to previously observed interactions; and (2) methods that predict interactions *ab initio*. For category (1), Deane *et al*. [12] and Deng *et al*. [13] introduced methods to tackle the problem of assigning reliabilities to interactions using similarity in mRNA expression profiles. Subsequently, Bader *et al*. [14] used additional features of interacting proteins, including functional similarity and high network clustering [15], to assign confidence scores to protein interactions. For category (2), Marcotte *et al*. [16], von Mering *et al*. [17], Myers *et al*. [18] and Jansen *et al*. [19] were among the first to predict new protein interactions by incorporating a combination of different features like high mRNA expression correlation, functional similarity, co-essentiality, and co-evolution. These schemes calculate a log-likelihood score for each interaction. As yet another approach in this category, Qi *et al*. [20] predicted new protein interactions using a method based on random forests. Presumably, the relative performance of each of these approaches versus the others involves a combination of factors such as the types of evidence used as inputs, the efficacy of each classification algorithms, and the sets of true and false interactions used as gold standards during training. Very recently, a second work by Qi *et al*. [21] studied the effect of the underlying classification algorithm by comparing the accuracies of different classifiers such as naïve Bayes, logistic regression, and decision trees.

Here, we perform a benchmarking analysis to evaluate the published interaction confidence schemes versus one another. Rather than isolate every factor that could influence a scheme's performance, we take a practical approach and evaluate the overall accuracy of each set of confidence scores as reported in the literature and available from the authors' websites. We limit ourselves to works that have assigned confidence scores to a common set of experimentally-observed interactions in yeast; this includes all of the category (1) schemes above, as well as the Qi. *et al*. scheme from category (2). The remaining *ab initio *schemes are concerned with predicting new interactions and do not assign confidences to those interactions that have already been experimentally observed. We also assess the performance of a "null hypothesis", a uniform scheme that considers the same probability for all interactions. To compare the quantitative accuracy of the methods, we examine the correlations between the confidence estimates and different biological attributes such as function and expression. As a further comparison criterion, we apply the signal processing concept of 'Signal-to-Noise Ratio' (SNR) to evaluate the significance of protein complexes identified in the network based on the different schemes [22]. The discovery of these complexes depends on the connectivity of the interaction network which, in turn, is influenced by the underlying interaction probabilities [22,23].

## Results

### Interaction confidence assignment schemes

Although large-scale protein interaction networks are being generated for a number of species, *S. cerevisiae *is perhaps the best studied among them and is associated with the largest variety and quantity of protein interaction data. Hence, most of the interaction probability schemes have been developed using the yeast protein interaction network as a guide. As the probability schemes were previously computed for different subsets of yeast protein-protein interactions, we compiled a test set of 11,883 yeast interactions common to all schemes. These yeast interactions were derived from both yeast two-hybrid [4,5] and mass-spectrometry-based [2,3] screens.

In total, we considered seven interaction probability assignment schemes, including Bader *et al*. [14] (2 schemes), Deane *et al*. [12], Deng *et al*. [13], Sharan *et al*. [23], Qi *et al*. [20] and a default scheme, where all interactions are assigned the same probability. Bader *et al*., Sharan *et al*. and Qi *et al*. have assigned specific probabilities to every yeast interaction, while Deane *et al*. and Deng *et al*. have grouped yeast interactions into high/medium/low confidence groups. All of the above schemes define and use some set of gold standard positive and negative interaction examples for the probability estimation.

#### Bader et al. (BADER_LOW/BADER_HIGH)

As a gold standard positive training data set, Bader *et al*. [14] used interactions determined by co-IP, in which the proteins were also one or two links apart in the Y2H network. The negative training data set was selected from interactions reported either by co-IP or Y2H, but whose distance (after excluding the interaction) was larger than the median distance in Y2H or co-IP respectively. Using these training data, they constructed a logistic regression model that computes the probability of each interaction based on explanatory variables including data source, number of interacting partners, and other topological features like network clustering. We refer to this scheme as Bader *et al*. (low) or BADER_LOW in our analysis.

Initially, the authors used measures based on Gene Ontology (GO) [24] annotations, co-expression, and presence of genetic interactions as measures to validate their data. However, they also combined these measurements into the probability score to bolster their confidence of true interactions. We consider these new confidence scores in our analysis as Bader *et al*. (high) or BADER_HIGH.

#### Deane et al. (DEANE)

Deane *et al*. [12] estimated the reliability of protein-protein interactions using the expression profiles of the interacting partners. Protein interactions observed in small-scale experiments that were also curated in the Database of Interacting Proteins (DIP) [25] were considered as the gold standard positive interactions. As a gold standard negative, they randomly picked protein pairs from the yeast proteome that were not reported in DIP. The authors used this information to compute the reliabilities of groups of interactions (obtained from an experiment or a database). Higher reliabilities were assigned to groups whose combined expression profile was closer to the gold standard positive than the gold standard negative interactions. Specifically, reliabilities were assigned to the whole DIP database, the set of all protein interactions generated in any high-throughput genome screen, and protein interactions generated by Ito *et al*. [4].

#### Deng et al. (DENG)

Deng *et al*. [13] estimated the reliabilities of different interaction data sources in a manner similar to Deane *et al*. [12]. They separately considered experiments that report pair-wise interactions like Y2H and those that report complex membership like mass spectrometry. Curated pair-wise interactions from the literature and membership in protein complexes from Munich Information center for Protein Sequences (MIPS) [26] were used as the gold standard positive set in each case. Randomly chosen protein pairs formed the gold standard negative data set. Reliabilities for each data source were computed using a maximum likelihood scheme based on the expression profiles of each data set. The authors evaluated reliabilities for Y2H data sources like Uetz *et al*. [5] and Ito *et al*. [4], and protein complex data sources like Tandem Affinity Purification (TAP) [2] and High-throughput Mass Spectrometric Protein Complex Identification (HMS-PCI) [3]. In addition to assigning reliabilities to each dataset, the authors also provided a conditional probability scheme to compute probabilities for groups of interactions observed in two or more data sources. This calculation results in assigning a high probability (0.99) to yeast interactions observed in more than 1 data source. We use the probabilities generated by this method for our comparative analysis.

#### Sharan et al. (SHARAN)

Recently, Sharan *et al*. [23] also implemented an interaction probability assignment scheme similar to the one proposed by Bader *et al*. The scheme assigned probabilities to interactions using a logistic regression model based on mRNA expression, interaction clustering and number of times an interaction was observed in independent experiments. Here, we use a modification of this scheme, assigning probabilities to interactions based only on direct experimental evidence. Specifically, interactions with at least two literature references or those that had a distance ≤ 2 in both the co-IP and Y2H networks were defined as the gold standard positives. Conversely, proteins at a distance > 4 in the entire network (after removing the interaction in question) were defined as the gold standard negatives. Binary variables were used to denote whether the interaction was reported in a co-IP data set, Y2H data set, a small-scale experiment or a large-scale experiment. Interaction probabilities were then estimated using logistic regression on the predictor parameters similarly to Bader *et al*. [14].

#### Qi et al. (QI)

In this study, the authors used interactions that were observed in small-scale experiments and reported by either DIP or Bader *et al*. as their gold standard positive training data [20]. Randomly picked protein pairs were used as the gold standard negative training data. The method incorporates direct evidence such as the type of experiment used to generate the data and indirect evidence like gene expression, existence of synthetic lethal interactions, and domain-domain interactions to construct a random forest (a collection of decision trees). The resulting forest is then used to calculate the probability that two proteins interact.

#### Equal probabilities (EQUAL)

Finally, we also considered the case in which all observed interactions were considered to be equally true. We refer to this case as EQUAL in the analysis.

A summary of all attributes used as inputs to the different probability schemes is provided in Table 1. It should be noted that even though the different probability schemes utilize some of the same types of inputs (e.g., experiment type, expression similarity), they may incorporate these inputs in different ways. For instance, both SHARAN and DENG use "experiment type" as input, but SHARAN explicitly includes each type of experiment as a separate indicator variable in its logistic regression function, while DENG pools data from each experimental type and assigns a single confidence level to the interactions in each pool.

We also compared global statistics such as the average and median probability assigned by each scheme (see Additional File 1). We found that most probability schemes had an average probability in the range of [0.3–0.5]. In contrast, Deane *et al*. (DEANE) had a very high average and median probability: over half of the interactions in the test set were assigned a probability of 1. We also computed Spearman correlations among the different probability schemes to measure their levels of inter-dependency (Table 2). The maximum correlation was seen between BADER_LOW and BADER_HIGH, as might be expected since both schemes were reported in the same study and BADER_HIGH was derived from BADER_LOW. On the other hand, Qi *et al*. (QI) had very low Spearman correlation with any of the probability schemes. The low correlation may reflect an inherent difference between schemes that assign probabilities to experimentally observed interactions and ones that predict protein interactions *ab initio*. The probabilities assigned by the schemes can be obtained from the Supplementary website [27].

### Quality assessment

One of the most objective ways to assess the performance of the different confidence assignment schemes would be to compare their success at correctly classifying a gold standard set of true protein interactions. However, all of the schemes considered in this analysis had already used the available gold standard sets of known yeast interactions in the training phase of their classifiers and, consequently, assigned high confidence scores to them. As an alternative approach, we employed five measures that had been shown to associate with true protein interactions [11,22,28,29] to gauge the performance of the schemes. One caveat of this approach is that, in some cases, one of the measures used to assess a scheme's performance had already been used (in full or in part) as an input to assigning its probabilities. To avoid circularity, this measure was used only for gauging the performance of the remaining schemes. For each of the five measures, two ways were used to estimate the level of association: Spearman correlation and weighted average (see Methods). Importantly, by using the Spearman correlation coefficient, we are in fact comparing how the schemes rank the interactions, not the absolute scores that are assigned. Note that the EQUAL probability scheme results in Spearman correlation of 0, by definition.

#### Presence of conserved interactions in other species

Presence of conserved interactions across species is believed to be associated with biologically meaningful interactions [29]. As our benchmark, we used yeast protein interactions that were conserved with measured *C. elegans *and *D. melanogaster *interactions obtained from the Database of Interacting Proteins (DIP). An interaction was considered conserved if homologs of the interacting yeast proteins were also interacting in another species. Homologs were based on amino-acid sequence similarity computed using BLAST [30], thus allowing a protein to possibly match with multiple proteins in the opposite species (if interacting yeast proteins were homologous to any pair of homologs with an observed interaction, the yeast interaction was counted as conserved). In particular, we allow interactions whose interacting proteins are themselves homologs, but filter cases where both the interacting proteins pointed to the same protein in the other species. We evaluated the weighted average and Spearman correlation between the probability assignment for each yeast interaction and the number of conserved interactions across worm and fly (0, 1, or 2). We used an E-value cut-off of 1 × 10^-10 ^to make the homology assignments (Table 3). We observed that SHARAN and BADER_HIGH had the highest weighted average and Spearman correlation. Not surprisingly, EQUAL had the lowest weighted average. Note that the conserved interactions test is a very strong filter for true interactions as it heavily depends on the level of completeness of the interaction networks of other species being considered. However, as the underlying set of interactions is the same across the different probability schemes, this filter affects all schemes similarly.

#### Expression correlation

Yeast expression data for ~790 conditions were obtained from the Stanford Microarray Database (SMD) [31]. For every pair of interacting proteins, we computed the Pearson correlation coefficient of expression. We then calculated the Spearman correlation and weighted average between the expression correlation coefficients of interacting proteins and their corresponding probability assignments in the different schemes (see Table 3 and Additional File 2). We found significant association between expression correlations and probabilities in the case of BADER_HIGH, BADER_LOW, QI and DENG. This result is expected as these schemes, with the exception of BADER_LOW, utilize expression similarity for interaction probability calculation. Surprisingly, DEANE probabilities showed very little correlation with expression, even though mRNA expression profiles were used as input in the prediction process reflecting the difference in the way expression similarity is incorporated in this method. In particular, DEANE is the only method where expression similarity between two interacting proteins is taken into account as the Euclidean distance between their expression profiles versus other methods which incorporated the Pearson correlation coefficient of expression. On the other hand, BADER_LOW had a higher Spearman correlation than SHARAN, though both had very similar weighted averages and did not utilize expression data in the training phase.

#### Gene Ontology (GO) similarity

As a first measure, we adopted the common notion that two interacting proteins are frequently involved in the same process and hence should have similar GO assignments [24]. The Gene Ontology terms are represented using a directed acyclic graph data structure in which an edge from term 'a' to term 'b' indicates that term 'b' is either a more specific functional type than term 'a', or is a part of term 'a'. As a result, terms that appear deeper in the graph are more specific. Moreover, specific terms also have fewer proteins assigned to them or their descendants.

Let "P_i_" and "P_j_" be two proteins that have been observed to interact with each other. To measure their functional similarity, we evaluated the size (number of proteins assigned to the term), represented as "S_ij_", of the deepest common GO term assignment (deepest common ancestor in graph) shared between them. Thus, a smaller value of S_ij _indicates a greater functional similarity between P_i _and P_j_. In addition, we also found that known yeast interactions generally have lower values for S_ij _than random background (see Additional File 3). To ensure that higher values of our GO measure correspond to higher performance (as is the case for other quality assessment metrics below), we use the negative of S_ij _(or -S_ij_) to represent the overall GO similarity.

Table 3 shows the relationship between GO similarity and the interaction probabilities for each scheme. Of the schemes that did not use functional annotations as inputs, DENG and SHARAN both had a very high Spearman correlation with GO (with DENG slightly higher than SHARAN). However, one potential concern was that GO functional assignments could incorporate evidence of co-expression which was used as an input by the DENG scheme. This potential circularity can be addressed by use of the partial correlation coefficient to factor out the dependency of GO on co-expression (see Additional File 4). However, the partial correlation is almost certainly an overcorrection since GO similarity and co-expression (and in fact any two lines of evidence) are expected to have some correlation if they are both predictive of true interactions. Regardless, with or without the correction, DENG and SHARAN scored within 2% of each other; thus the two schemes are practically indistinguishable by the GO metric.

#### Signal-to-noise ratio of protein complexes

Most cellular processes involve proteins that act together by assembling into functional complexes. Several methods [23,32-35] have been developed to identify complexes embedded within a protein interaction network, in which a complex is typically modeled as a densely-connected protein sub-network. Recently, we showed that the quality of these identified protein complexes could be estimated by computing their signal-to-noise ratio (SNR), a standard measure used in information theory and signal processing to assess data quality (see Methods) [22]. Essentially, SNR evaluates the density of complexes found in the protein interaction network against a randomized version of the same network.

As the SNR is independent of the number of complexes reported, its value can be directly compared across the different probability schemes. For discovery of protein complexes, we applied a previously-published algorithm [23] which includes interaction probabilities in the complex identification process. SNR was then computed on the set of complexes identified by each probability scheme. Results are shown in Table 4; out of all of the schemes, DENG had the highest SNR of protein complex detection.

#### Conservation rate coherency

Interacting proteins have been shown to evolve at similar rates, probably due to selection pressure to maintain the interaction over time [28]. For every pair of interacting proteins, P_i _and P_j_, let "r_i_" and "r_j_" be their respective rates of evolution. We then computed a "conservation rate coherency score" (CR_ij_) as the negative absolute value of the difference between the evolutionary rates of the two corresponding genes: CR_ij _= -| r_i _- r_j _|. The negative absolute value was used to ensure that higher values represent higher performance, consistent with other metrics.

Evolutionary rates were obtained from Fraser *et al*. [36] and estimated using nucleotide substitution frequencies. We calculated the Spearman correlation between the values of CR for the interacting proteins and their corresponding probability assignments in the different schemes (see Table 4). For all probability assignment schemes we obtained a statistically significant correlation (p-value < 0.05) between the conservation rate coherency scores and the corresponding probabilities, indicating that proteins with high probability interactions tend to have similar conservation rates. The highest correlation was obtained for DENG.

## Discussion

A brief review of the performance results suggests that the DENG method (Deng *et al*.) emerges as the clear winner, with top scores in three out of four non-circular quality metrics. Comprising a 'second tier' are BADER_HIGH, BADER_LOW (the two Bader methods) and SHARAN, which perform very similarly across most metrics with some differences in conservation coherency or gene expression (for which SHARAN performs better or worse, respectively). BADER_LOW, which considers experiment type and interaction clustering as inputs, has a higher expression score than SHARAN, which considers experiment type only, implying that interaction clustering helps capture expression similarity. Interestingly, BADER_HIGH, which incorporates more input attributes than BADER_LOW or SHARAN, does not have substantially higher rankings. Thus, in this case, adding more inputs to a probability assignment scheme does not appear to strongly enhance its quality.

As for the remaining schemes with lower overall performance (DEANE and QI), it is interesting to note that these were arguably the least and most sophisticated schemes, respectively. The DEANE method relied on only a single evidence type for assigning confidences, that of gene expression, whereas it appears that other factors may have been more informative (Table 1). In contrast to DEANE, QI had the largest number of inputs for assigning confidences and, among these, included data on both co-expression and experiment type. However, it is well known that classifier accuracy can be degraded by including many irrelevant input variables [37], and perhaps this is the reason for QI's lower performance. As an alternative explanation, in Qi *et al*.'s evaluation of classification schemes, they concluded that their method was very successful in predicting co-complex membership, but performed poorly when considering physical interactions [21]. In our analysis, all interactions (even co-complex membership) were treated as pair-wise protein interactions, and this assumption may have contributed to the poor performance of Qi *et al*. Certainly, their classification method was among the most sophisticated of the schemes that we evaluated, and as such it is worthy of future exploration (perhaps with different sources of input data) regardless of its performance in the present study.

Finally, EQUAL almost always scored lowest, regardless of quality metric. Thus, utilizing any probability scheme is better than considering all observed interactions to be true or equally probable.

Beyond these broad rankings, is it possible to synthesize data from five largely independent metrics to arrive at an overall quantitative index of performance? As one approach, we normalized the scores for each metric as a fraction of the best score achieved within that metric over all confidence assignment schemes (i.e., for each metric, the highest score was fixed to 1 and the scores of the remaining schemes were converted to fractional values between 0 and 1). Table 5 summarises the fractional scores for the six probability schemes and five quality assessment measures. Note that expressing scores as fractional values is an intermediate normalization which preserves the score distribution but compresses its range; although potentially more informative than the non-parametric analysis above based only on ranks, it must also be interpreted with more caution. However, in this case, the fractional scores reinforce the findings reported above based on rank.

## Conclusion

We have compared and contrasted seven probability assignment schemes for yeast protein interactions. Surprisingly, Deng *et al*. performs significantly better than others while being one of the least sophisticated. It assigns discrete probability scores to large groups of interactions rather than to individuals, and it inputs just two lines of evidence, experiment type and expression similarity, rather than many. Generalizing these observations, more complex approaches are so far unable to outperform simpler variants. Thus, we arrive at a somewhat unexpected conclusion: At least in interaction confidence assignment, sometimes less means more.

## Methods

### GO databases

The Gene Ontology annotations for yeast proteins were obtained from the July 5^th^, 2005 download of the Saccharomyces Genome Database (SGD) [38]; the graph of relations between terms was obtained from the Gene Ontology consortium .

### Weighted average

The weighted average is given by WA=∑i=1Npi∗mi∑i=1Npi
 MathType@MTEF@5@5@+=feaafiart1ev1aaatCvAUfKttLearuWrP9MDH5MBPbIqV92AaeXatLxBI9gBaebbnrfifHhDYfgasaacH8akY=wiFfYdH8Gipec8Eeeu0xXdbba9frFj0=OqFfea0dXdd9vqai=hGuQ8kuc9pgc9s8qqaq=dirpe0xb9q8qiLsFr0=vr0=vr0dc8meaabaqaciaacaGaaeqabaqabeGadaaakeaacqWGxbWvcqWGbbqqcqGH9aqpdaWcaaqaamaaqahabaGaemiCaa3aaSbaaSqaaiabdMgaPbqabaaabaGaemyAaKMaeyypa0JaeGymaedabaGaemOta4eaniabggHiLdGccqGHxiIkcqWGTbqBdaWgaaWcbaGaemyAaKgabeaaaOqaamaaqahabaGaemiCaa3aaSbaaSqaaiabdMgaPbqabaaabaGaemyAaKMaeyypa0JaeGymaedabaGaemOta4eaniabggHiLdaaaaaa@472B@, where *p*_*i *_is the probability of a given interaction and *m*_*i *_is the value of one of the five measures for the interaction.

### Signal to noise ratio (SNR)

To compute SNR, a search for dense interaction complexes is initiated from each node (protein) and the highest scoring complex from each is reported. This yields a distribution of complex scores over all nodes in the network. A score distribution is also generated for 100 randomized networks, which have identical degree distribution to the original network. SNR is computed using these original and random score distributions (representing signal and noise, respectively) according to the standard formula [39] using the root-mean-square (rms):

SNR=log⁡10rms(original complex scores)rms(random complex scores),where rms(x1⋯xM)=1M∑i=1Mxi2
 MathType@MTEF@5@5@+=feaafiart1ev1aaatCvAUfKttLearuWrP9MDH5MBPbIqV92AaeXatLxBI9gBaebbnrfifHhDYfgasaacH8akY=wiFfYdH8Gipec8Eeeu0xXdbba9frFj0=OqFfea0dXdd9vqai=hGuQ8kuc9pgc9s8qqaq=dirpe0xb9q8qiLsFr0=vr0=vr0dc8meaabaqaciaacaGaaeqabaqabeGadaaakeaafaqabeqacaaabaGaee4uamLaeeOta4KaeeOuaiLaeyypa0JagiiBaWMaei4Ba8Maei4zaC2aaSbaaSqaaiabigdaXiabicdaWaqabaGcdaWcaaqaaiabbkhaYjabb2gaTjabbohaZjabcIcaOiabb+gaVjabbkhaYjabbMgaPjabbEgaNjabbMgaPjabb6gaUjabbggaHjabbYgaSjabbccaGiabbogaJjabb+gaVjabb2gaTjabbchaWjabbYgaSjabbwgaLjabbIha4jabbccaGiabbohaZjabbogaJjabb+gaVjabbkhaYjabbwgaLjabbohaZjabcMcaPaqaaiabbkhaYjabb2gaTjabbohaZjabcIcaOiabbkhaYjabbggaHjabb6gaUjabbsgaKjabb+gaVjabb2gaTjabbccaGiabbogaJjabb+gaVjabb2gaTjabbchaWjabbYgaSjabbwgaLjabbIha4jabbccaGiabbohaZjabbogaJjabb+gaVjabbkhaYjabbwgaLjabbohaZjabcMcaPaaacqGGSaalaeaacqqG3bWDcqqGObaAcqqGLbqzcqqGYbGCcqqGLbqzcqqGGaaicqqGYbGCcqqGTbqBcqqGZbWCdaqadiqaaiabdIha4naaBaaaleaacqaIXaqmaeqaaOGaeS47IWKaemiEaG3aaSbaaSqaaiabd2eanbqabaaakiaawIcacaGLPaaaaaGaeyypa0ZaaOaaaeaadaWcaaqaaiabigdaXaqaaiabd2eanbaadaaeWbqaaiabdIha4naaDaaaleaacqWGPbqAaeaacqaIYaGmaaaabaGaemyAaKMaeyypa0JaeGymaedabaGaemyta0eaniabggHiLdaaleqaaaaa@A0D3@

where *M *denotes the total number of complexes (in this case, equal to the number of nodes) and *x*_*i *_represents the score of an individual complex.

## Authors' contributions

The conception and design of this study was done by SS, TI and RS. The conservation coherency calculations were carried out by TS and the calculations for other metrics were done by SS. The drafts of the manuscript were prepared by SS and TI. The revisions and critical analysis of the manuscript were carried out by RS, ER and TS. All authors read and approved the final manuscript.

## Supplementary Material

Additional File 1 Global properties of the probability assignment schemes. Shows properties like average and median probabilities. Click here for file

Additional File 2 Correlation of interaction probabilities with mRNA expression correlation. Ribosomal components are among the most co-expressed genes, and could potentially lead to the observed relative importance of co-expression data. To check for the effect of ribosomal proteins, we filtered the yeast interaction set in our analysis to remove all ribosomal proteins and calculated the correlation between co-expression and interaction probability. These results are shown in Additional Table 2. The numbers in the brackets represent the values of Spearman correlation coefficient and weighted average after removing the ribosomal proteins from the interaction data. We observe that removing the ribosomal proteins does not change the values significantly. Click here for file

Additional File 3 Histograms of GO similarity scores. We evaluated the GO similarity scores for known yeast interactions reported in the MIPS database [26]. The histogram of the scores is shown in Additional Figure 1A. We also generated a background distribution by computing the GO similarity scores for 1,000 random interactions (Additional Figure 1B). These random interactions were generated by picking pairs of proteins randomly from the set of interacting proteins in yeast. It is evident from the two figures that true proteins interactions (i.e known yeast interactions reported in MIPS) generally have lower GO similarity scores than the background. Click here for file

Additional File 4 Spearman partial correlations for schemes using expression as input. **Spearman Partial Rank Correlation Coefficient**. The Spearman partial rank correlation coefficient between two random variables A and X, given the fact that both A and X are correlated to random variable Y, denotes the correlation between A and X, when Y is kept constant. It is calculated as follows: rAX,Y=rAX−rXYrAY(1−rXY2)(1−rAY2)
 MathType@MTEF@5@5@+=feaafiart1ev1aaatCvAUfKttLearuWrP9MDH5MBPbIqV92AaeXatLxBI9gBaebbnrfifHhDYfgasaacH8akY=wiFfYdH8Gipec8Eeeu0xXdbba9frFj0=OqFfea0dXdd9vqai=hGuQ8kuc9pgc9s8qqaq=dirpe0xb9q8qiLsFr0=vr0=vr0dc8meaabaqaciaacaGaaeqabaqabeGadaaakeaacqWGYbGCdaWgaaWcbaGaemyqaeKaemiwaGLaeiilaWIaemywaKfabeaakiabg2da9maalaaabaGaemOCai3aaSbaaSqaaiabdgeabjabdIfaybqabaGccqGHsislcqWGYbGCdaWgaaWcbaGaemiwaGLaemywaKfabeaakiabdkhaYnaaBaaaleaacqWGbbqqcqWGzbqwaeqaaaGcbaWaaOaaaeaacqGGOaakcqaIXaqmcqGHsislcqWGYbGCdaqhaaWcbaGaemiwaGLaemywaKfabaGaeGOmaidaaOGaeiykaKIaeiikaGIaeGymaeJaeyOeI0IaemOCai3aa0baaSqaaiabdgeabjabdMfazbqaaiabikdaYaaakiabcMcaPaWcbeaaaaaaaa@51B7@ Here, r_AX_, r_XY _and r_AY _represent the Spearman correlation coefficients between A and X, X and Y, and, A and Y respectively. The significance level is given by DAX,Y=1/2N−4ln⁡(1+rAX,Y1−rAX,Y)
 MathType@MTEF@5@5@+=feaafiart1ev1aaatCvAUfKttLearuWrP9MDH5MBPbIqV92AaeXatLxBI9gBaebbnrfifHhDYfgasaacH8akY=wiFfYdH8Gipec8Eeeu0xXdbba9frFj0=OqFfea0dXdd9vqai=hGuQ8kuc9pgc9s8qqaq=dirpe0xb9q8qiLsFr0=vr0=vr0dc8meaabaqaciaacaGaaeqabaqabeGadaaakeaacqWGebardaWgaaWcbaGaemyqaeKaemiwaGLaeiilaWIaemywaKfabeaakiabg2da9iabigdaXiabc+caViabikdaYmaakaaabaGaemOta4KaeyOeI0IaeGinaqdaleqaaOGagiiBaWMaeiOBa42aaeWaceaadaWcaaqaaiabigdaXiabgUcaRiabdkhaYnaaBaaaleaacqWGbbqqcqWGybawcqGGSaalcqWGzbqwaeqaaaGcbaGaeGymaeJaeyOeI0IaemOCai3aaSbaaSqaaiabdgeabjabdIfayjabcYcaSiabdMfazbqabaaaaaGccaGLOaGaayzkaaaaaa@4D61@ D_AX, Y _has a normal distribution with zero mean and variance one. N represents the size of the data set. Click here for file

## Figures and Tables

**Table 1:** Summary of input attributes for the different probability schemes.

**Prob. Scheme**	**Experiment Type**	**Number of Experimental Observations**	**Protein-DNA binding**	**Gene/Protein Expression**	**Interaction Clustering**	SL*	GO*	DDI*	Gene Fusion/Co-occur/Nbrhd*
**BADER_LOW**	X	X			X				
**BADER_HIGH**	X	X		X	X	X	X		
**DEANE**		X		X					
**DENG**	X	X		X					
**SHARAN**	X	X							
**QI**	X		X	X	X	X	X	X	X
**EQUAL**									

*SL: Synthetic Lethal; GO: Gene Ontology; DDI: Domain-domain Interactions; Nbrhd: Neighborhood

**Table 2:** Correlation of different probability schemes*.

	**BADER_HIGH**	**DEANE**	**DENG**	**SHARAN**	**QI**
**BADER_LOW**	0.923	0.655	0.633	0.626	0.095
	**BADER_HIGH**	0.672	0.644	0.665	0.151
		**DEANE**	0.718	0.847	-0.090
			**DENG**	0.680	0.185
				**SHARAN**	-0.013

*p-values of all correlation measurements were significant (*p*-value ≤ 2 × 10^-16^).

**Table 3:** Correlation of interaction probabilities with the GO similarity measure, mRNA expression correlation and interaction conservation.*

**Prob. Scheme**	**GO Annotation**		**Expression Correlation**		**Interaction Conservation**	
	
	**SC**	**WA**	**SC**	**WA**	**SC**^#^	**WA**^#^
**BADER_LOW**	0.424	-5.850	**0.185**	**0.494**	0.132	0.147
**BADER_HIGH**	*0.501*	*-5.680*	*0.223*	*0.503*	**0.136**	**0.158**
**DEANE**	0.385	-5.910	*0.016*	*0.481*	0.098	0.139
**DENG**	**0.490**	**-5.620**	*0.185*	*0.511*	0.102	0.147
**SHARAN**	**0.471**	**-5.710**	0.050	0.492	**0.134**	**0.158**
**QI**	*0.425*	*-6.040*	*0.269*	*0.495*	0.080	0.125
**EQUAL**	--	-6.320	--	0.482	--	0.102

*Bold values indicate the scheme that performs the best. Italicized values indicate potential circularity, i.e., schemes that use GO annotations or mRNA expression profiles for confidence scoring that are similar to those used here for comparative assessment. *P*-values for all the Spearman correlation measurements are significant. SC: Spearman Correlation; WA: Weighted Average.

^# ^All measurements were done at an E-value cut-off of 1 × 10^-10^.

**Table 4:** Associations of conservation rate coherency scores and SNR with interaction probabilities.

**Prob. Scheme**	Conservation Coherency (SC*)	**SNR**
**BADER_LOW**	0.090	0.734
**BADER_HIGH**	0.104	0.735
**DEANE**	0.113	0.537
**DENG**	**0.141**	**0.950**
**SHARAN**	0.126	0.742
**QI**	0.080	0.706
**EQUAL**	--	0.657

* SC: Spearman Correlation. Bold values indicate the scheme which performs the best. Note that conservation scores based on weighted averages were omitted as they were very similar across the different confidence assignment schemes.

**Table 5:** Fractional scores of the confidence assignment schemes in each of the five quality measures*.

**Probability Scheme**	**Gene Ontology **(SC)	**Interaction Conservation **(SC at 1 × 10^-10^)	**Gene Expression **(SC)	**SNR**	**Conservation Coherency **(SC)
**DENG: **Deng *et al*.	**1.00**	0.76	--	**1.00**	**1.00**
**BADER_HIGH: **Bader *et al*. (high)	--	**1.00**	--	0.77	0.74
**BADER_LOW: **Bader *et al*. (low)	0.86	0.98	**1.00**	0.77	0.64
**SHARAN: **Sharan *et al*.	**0.96**	**1.00**	0.27	0.78	0.89
**DEANE: **Deane *et al*.	0.78	0.73	--	0.57	0.80
**QI: **Qi *et al*.	--	0.58	--	0.74	0.57

*Fractional scores are between [0,1] with 1 performing the best (indicated in bold for each measure). Cells with a dash (-) indicate circularity, i.e., the measures used as (full or partial) input to the corresponding probability schemes. SC: Spearman Correlation; SNR: Signal to Noise Ratio.
