# Supplementary material for: A direct comparison of protein interaction confidence assignment schemes
Source: BMC Bioinformatics. 2006 Jul 26;7:360. doi: 10.1186/1471-2105-7-360 (PMC1550431; doi:10.1186/1471-2105-7-360)
Supplement: Additional File 1 — Global properties of the probability assignment schemes. Shows properties like average and median probabilities. [file 1471-2105-7-360-S1.doc]

## Additional Table 1 - Global properties of the probability assignment schemes.

| **Prob. Scheme** | **Average Probability** | **Median Probability** | **# Intrxns. with**  **Prob. ≥ 0.5** |
| --- | --- | --- | --- |
| **BADER_LOW** | 0.51 | 0.55 | 6,886 |
| **BADER_HIGH** | 0.48 | 0.50 | 5,896 |
| **DEANE** | 0.72 | 1.00 | 7,531 |
| **DENG** | 0.39 | 0.25 | 4,799 |
| **SHARAN** | 0.38 | 0.42 | 1,121 |
| **QI** | 0.46 | 0.47 | 4929 |
| **EQUAL** | 0.99 | 0.99 | 11,883 |
