# Supplementary material for: A direct comparison of protein interaction confidence assignment schemes
Source: BMC Bioinformatics. 2006 Jul 26;7:360. doi: 10.1186/1471-2105-7-360 (PMC1550431; doi:10.1186/1471-2105-7-360)
Supplement: Additional File 2 — Correlation of interaction probabilities with mRNA expression correlation. Ribosomal components are among the most co-expressed genes, and could potentially lead to the observed relative importance of co-expression data. To check for the effect of ribosomal proteins, we filtered the yeast interaction set in our analysis to remove all ribosomal proteins and calculated the correlation between co-expression and interaction probability. These results are shown in Additional Table 2. The numbers in the brackets represent the values of Spearman correlation coefficient and weighted average after removing the ribosomal proteins from the interaction data. We observe that removing the ribosomal proteins does not change the values significantly. [file 1471-2105-7-360-S2.doc]

## Additional Table 2 - Correlation of interaction probabilities with mRNA expression correlation. ^†^

| **Prob. Scheme** | **Expression Correlation** | |
| --- | --- | --- |
|  | **SC** | **WA** |
| **BADER_LOW** | **0.185 (0.187)** | **0.494 (0.497)** |
| **BADER_HIGH** | *0.223 (0.221)* | *0.503 (0.505)* |
| **DEANE** | *0.016 (0.010)* | *0.481 (0.483)* |
| **DENG** | *0.185 (0.185)* | *0.511 (0.514)* |
| **SHARAN** | 0.050 (0.045) | 0.492 (0.495) |
| **QI** | *0.269 (0.274)* | *0.495 (0.499)* |
| **EQUAL** | — | 0.482 (0.485) |

^†^The values in brackets correspond to the yeast interaction set which is filtered for ribosomal proteins.

*Bold values indicate the scheme that performs the best. Italicized values indicate potential circularity, i.e., schemes that use mRNA expression profiles for confidence scoring that are similar to those used here for comparative assessment. *P*-values for all the Spearman correlation measurements are significant. SC: Spearman Correlation; WA: Weighted Average.
