# Supplementary material for: A direct comparison of protein interaction confidence assignment schemes
Source: BMC Bioinformatics. 2006 Jul 26;7:360. doi: 10.1186/1471-2105-7-360 (PMC1550431; doi:10.1186/1471-2105-7-360)
Supplement: Additional File 3 — Histograms of GO similarity scores. We evaluated the GO similarity scores for known yeast interactions reported in the MIPS database [26]. The histogram of the scores is shown in Additional Figure 1A. We also generated a background distribution by computing the GO similarity scores for 1,000 random interactions (Additional Figure 1B). These random interactions were generated by picking pairs of proteins randomly from the set of interacting proteins in yeast. It is evident from the two figures that true proteins interactions (i.e known yeast interactions reported in MIPS) generally have lower GO similarity scores than the background. [file 1471-2105-7-360-S3.doc]

## Additional Figure 1

## (A) Histogram of GO similarity scores for the known yeast interactions reported in the MIPS database.


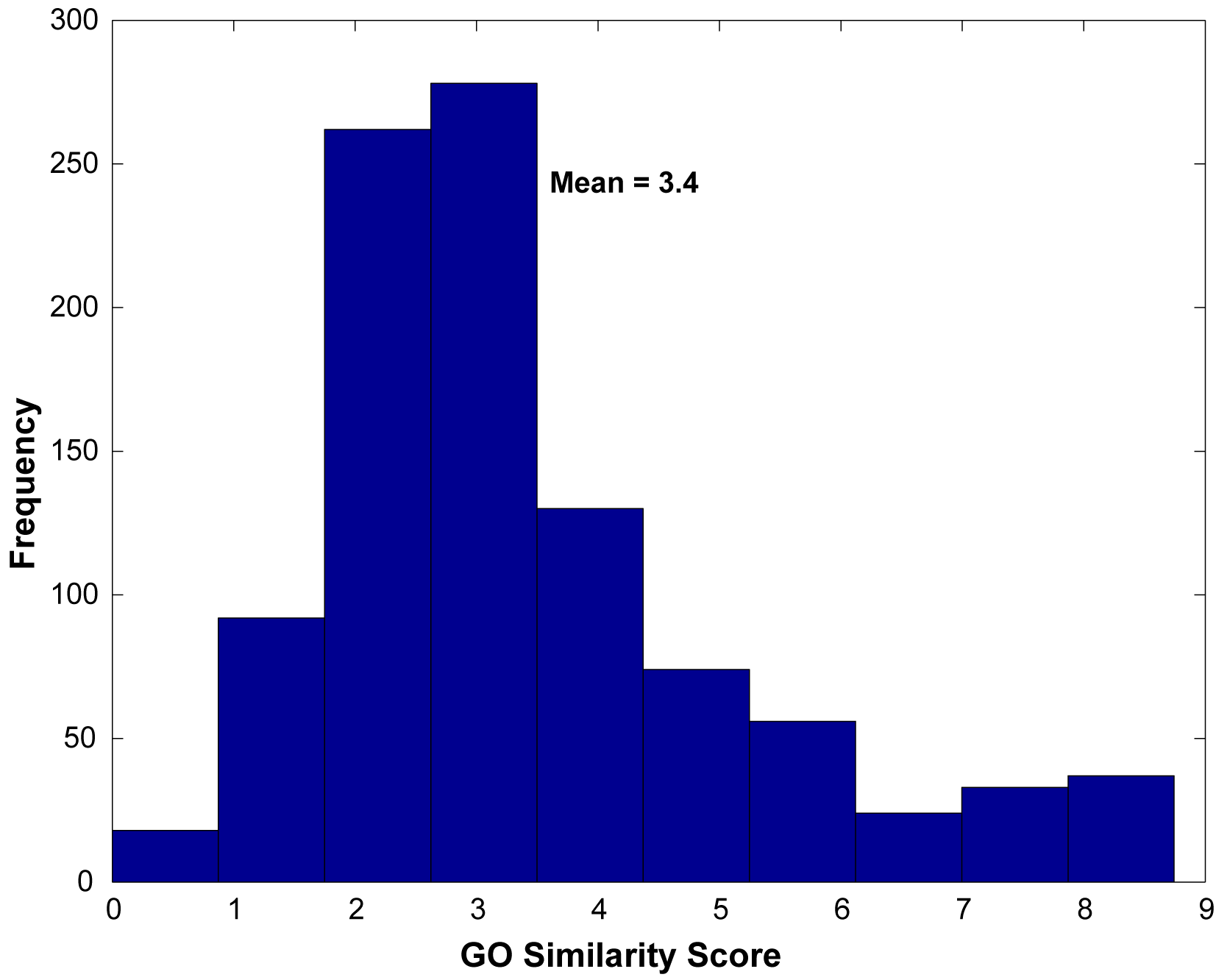


**(B) Histogram of GO similarity scores for 1,000 interactions generated randomly.**

**
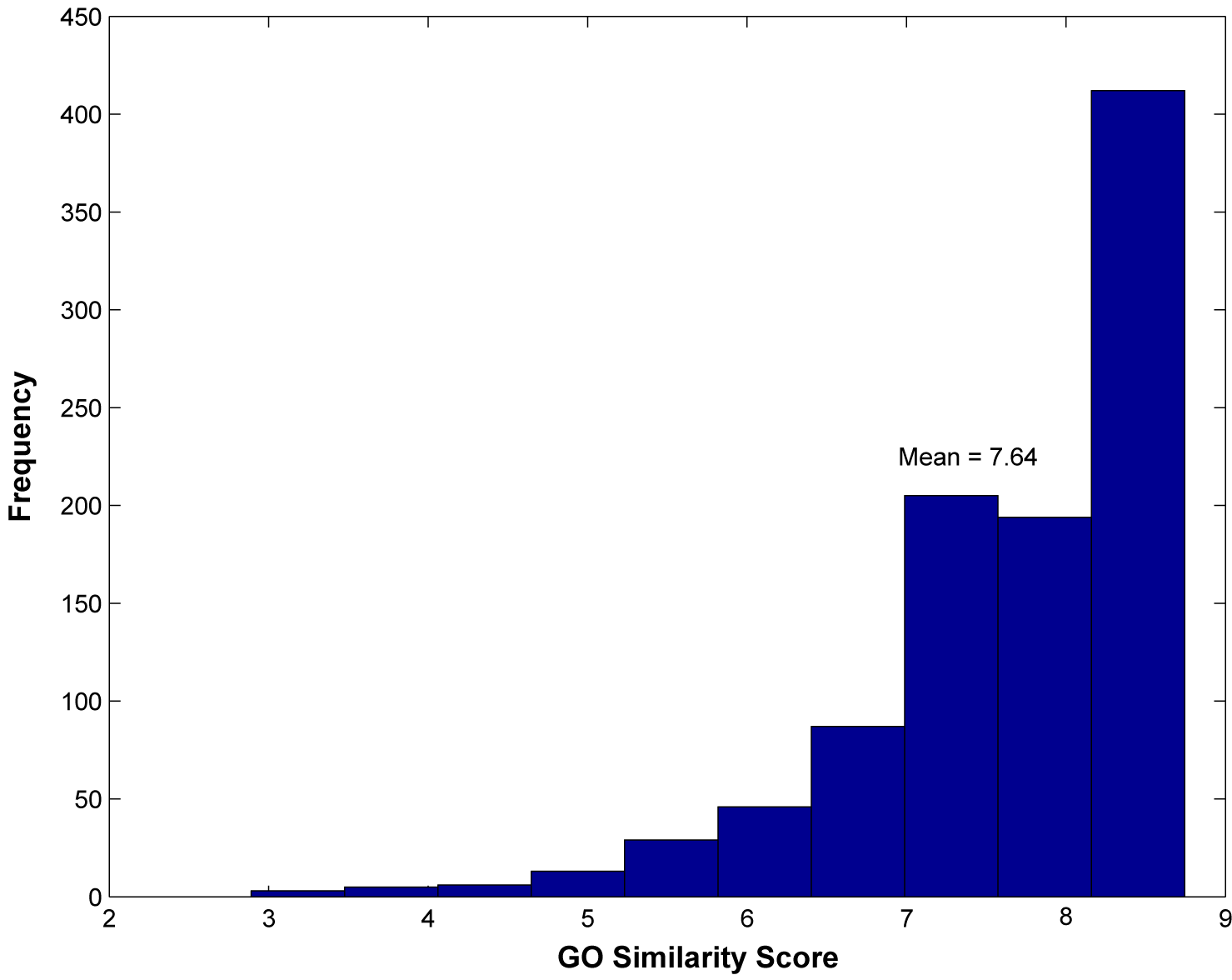
**
