# Supplementary material for: A direct comparison of protein interaction confidence assignment schemes
Source: BMC Bioinformatics. 2006 Jul 26;7:360. doi: 10.1186/1471-2105-7-360 (PMC1550431; doi:10.1186/1471-2105-7-360)
Supplement: Additional File 4 — Spearman partial correlations for schemes using expression as input. Spearman Partial Rank Correlation Coefficient. The Spearman partial rank correlation coefficient between two random variables A and X, given the fact that both A and X are correlated to random variable Y, denotes the correlation between A and X, when Y is kept constant. It is calculated as follows: rAX,Y=rAX−rXYrAY(1−rXY2)(1−rAY2) MathType@MTEF@5@5@+=feaafiart1ev1aaatCvAUfKttLearuWrP9MDH5MBPbIqV92AaeXatLxBI9gBaebbnrfifHhDYfgasaacH8akY=wiFfYdH8Gipec8Eeeu0xXdbba9frFj0=OqFfea0dXdd9vqai=hGuQ8kuc9pgc9s8qqaq=dirpe0xb9q8qiLsFr0=vr0=vr0dc8meaabaqaciaacaGaaeqabaqabeGadaaakeaacqWGYbGCdaWgaaWcbaGaemyqaeKaemiwaGLaeiilaWIaemywaKfabeaakiabg2da9maalaaabaGaemOCai3aaSbaaSqaaiabdgeabjabdIfaybqabaGccqGHsislcqWGYbGCdaWgaaWcbaGaemiwaGLaemywaKfabeaakiabdkhaYnaaBaaaleaacqWGbbqqcqWGzbqwaeqaaaGcbaWaaOaaaeaacqGGOaakcqaIXaqmcqGHsislcqWGYbGCdaqhaaWcbaGaemiwaGLaemywaKfabaGaeGOmaidaaOGaeiykaKIaeiikaGIaeGymaeJaeyOeI0IaemOCai3aa0baaSqaaiabdgeabjabdMfazbqaaiabikdaYaaakiabcMcaPaWcbeaaaaaaaa@51B7@ Here, rAX, rXY and rAY represent the Spearman correlation coefficients between A and X, X and Y, and, A and Y respectively. The significance level is given by DAX,Y=1/2N−4ln⁡(1+rAX,Y1−rAX,Y) MathType@MTEF@5@5@+=feaafiart1ev1aaatCvAUfKttLearuWrP9MDH5MBPbIqV92AaeXatLxBI9gBaebbnrfifHhDYfgasaacH8akY=wiFfYdH8Gipec8Eeeu0xXdbba9frFj0=OqFfea0dXdd9vqai=hGuQ8kuc9pgc9s8qqaq=dirpe0xb9q8qiLsFr0=vr0=vr0dc8meaabaqaciaacaGaaeqabaqabeGadaaakeaacqWGebardaWgaaWcbaGaemyqaeKaemiwaGLaeiilaWIaemywaKfabeaakiabg2da9iabigdaXiabc+caViabikdaYmaakaaabaGaemOta4KaeyOeI0IaeGinaqdaleqaaOGagiiBaWMaeiOBa42aaeWaceaadaWcaaqaaiabigdaXiabgUcaRiabdkhaYnaaBaaaleaacqWGbbqqcqWGybawcqGGSaalcqWGzbqwaeqaaaGcbaGaeGymaeJaeyOeI0IaemOCai3aaSbaaSqaaiabdgeabjabdIfayjabcYcaSiabdMfazbqabaaaaaGccaGLOaGaayzkaaaaaa@4D61@ DAX, Y has a normal distribution with zero mean and variance one. N represents the size of the data set. [file 1471-2105-7-360-S4.doc]

**Additional Table 3 – Effect of correlation between GO and expression.**

| **Prob Scheme** | **GO (SpC)** |
| --- | --- |
| **DEANE** | 0.383 |
| **DENG** | 0.451 |

* SpC = Spearman partial correlation. Both schemes used expression as input to assign confidence scores to protein interactions.
